# Immunological Features and Clinical Benefits of Conjugate Vaccines against Bacteria

**DOI:** 10.1155/2015/934504

**Published:** 2015-08-18

**Authors:** Paolo Durando, Saul N. Faust, Antoni Torres

**Affiliations:** ^1^Department of Health Sciences, School of Medical and Pharmaceutical Sciences, Postgraduate School in Occupational Medicine, University of Genoa and Medical Surveillance Unit for Health Care Workers, Occupational Medicine Unit, IRCCS San Martino Hospital, Largo R. Benzi 10, 16132 Genoa, Italy; ^2^NIHR Wellcome Trust Clinical Research Facility, University of Southampton, C Level, West Wing, Mailpoint 218, UK; ^3^University Hospital Southampton NHS Foundation Trust, Tremona Road, Southampton SO16 6YD, UK; ^4^Pneumology Department, Clinic Institute of Thorax, Hospital Clinic of Barcelona, Institut d'Investigacions Biomediques August Pi i Sunyer (IDIBAPS), University of Barcelona, Ciber de Enfermedades Respiratorias (CIBERES), Villarroel 170, 08036 Barcelona, Spain

Bacteria such as* Streptococcus pneumoniae*,* Neisseria meningitidis*, and* Haemophilus influenzae* are important pathogens that cause invasive and noninvasive diseases with a still high burden in terms of both morbidity and mortality worldwide [1–4].

The cornerstone for the prevention of these pathologies is by vaccination. In the last decades, significant advancement in the knowledge concerning both the mechanisms of pathogenicity of these pathogens, at a molecular level, and the features of the immune response to natural infection and vaccines have been achieved in humans, thanks to converging approaches of different disciplines, ranging from pathology to microbiology, immunology, vaccinology, and omics sciences (such as genomics and proteomics).

The acquisition of this knowledge is also of particular importance for public health policy makers, in order to establish new vaccines into clinical practice using well-designed immunization strategies.

First generation vaccines were based on bacterial capsular polysaccharides; yet, most of these antigens are considered T-independent antigens, showing significant gaps in terms of immunogenicity, particularly with respect to the generation of the immune memory [5–7].

The development of protein-polysaccharide conjugation technology in the 1980s allowed the availability of novel vaccines against* Haemophilus influenzae* type b (Hib) and different serogroups of* Neisseria meningitidis* [8, 9] that have demonstrated a very good safety and tolerability, together with the capability of eliciting a strong immunogenicity combined with the demonstration of the anamnestic antibody responses.

The main advantages of the conjugation technology used in bacterial vaccines, due to the generation of a T cell-dependent immune response, are briefly outlined:

- Improvement of the priming: immunogenic also in infants and young children (Ab-response, predominantly of the IgG1 isotype).
- Capability of eliciting an immunogenic memory response (production of long-lived memory B-cells) and a booster effect upon new contact with the specific antigen (revaccination).
- Capability of leading to affinity maturation of the Ab-response, with a consequent increased Ab-ag fit and improved opsonising function.
- Generation of a mucosal immune response (secretory IgA and mucosally active IgG).
- Reduction of the mucosal carriage (a prerequisite of herd protection).

Since the implementation of the Hib conjugate vaccines [10] and their successful introduction into the paediatric immunization programme of some countries in the early 1990s, with the near elimination of Hib meningitis [11–13], it was clear that this was only the pivot of a series of successful experiences against other bacterial species relevant to public health globally.

The demonstration of the effectiveness of the immunization programs in children with these new generation vaccines was the direct consequence of their good immunological characteristics [14]. The implementation of safe and effective meningococcal type C (MenC) vaccines followed Hib vaccine programmes, with subsequent heptavalent pneumococcal conjugate vaccine (PCV7) from the mid 2000s, and further formulations expanding the antigens coverage (i.e., Men AC, Men ACW135Y, PCV10, and PCV13) [15–19].

These vaccines have proven effective for fighting not only invasive diseases, such as sepsis and meningitis, but also other important noninvasive diseases, such as community acquired pneumonia and acute otitis media in both children and adults, with new interesting perspectives for optimizing current prevention strategies in the future [20–22].

The herd protection observed among unimmunized populations living in countries where routine vaccination programs were initially implemented was due to the indirect effect of vaccination on nasopharyngeal carriage of the bacteria in healthy carriers. The radical change of their epidemiological and ecological pictures exemplified a further unanticipated positive impact of the wide use of these conjugate vaccines, further stressing how precious they were to obtain the control of the related diseases among the entire population [19, 23].

With respect to the very new and recently licensed meningococcal type B vaccine, a multicomponent approach to its development was used: efforts have been made to identify key-protein antigens capable of preventing Men B infection and associated invasive disease and possibly those sustained by other meningococcal serogroups too [24–27]. Whether new meningitis B vaccines can also provide population immunity remains to be seen.

Available evidence indicates that a majority of childhood meningitis mortality is preventable with existing Hib and PCV vaccines and these findings are consistent with the other empirical evidence and reviews [28]. The same can be extrapolated for the different available types of meningococcal vaccines (Men C, Men ACW135Y, and Men b) in Europe, depending on the different geographical area [29].

We hope that readers can appreciate the aim of this special issue of stimulating the continuing efforts within the scientific community in order to (i) understand the immunological interactions between conjugate and/or the other novel vaccine technology and the human host, (ii) develop novel immunization strategies for improving the prevention of* Streptococcus pneumoniae* and* Neisseria meningitidis* related conditions, and (iii) evaluate the conjugate vaccines use, particularly in terms of efficacy and effectiveness.

Immunologists, vaccinologists, microbiologists, together with paediatricians, infectious diseases specialists, and pulmonologists, general practitioners, public health experts, and policy makers could be mainly interested in the contents of the papers included in it.
